# A cross-sectional study investigating the association between parental daily brushing, extended breastfeeding, or parental smoking habit and early childhood dental caries in 4-year-old children: the Japan Environment and Children’s Study

**DOI:** 10.1186/s12887-025-05997-8

**Published:** 2025-08-08

**Authors:** Yuichiro Miura, Tomohisa Suzuki, Keita Kanamori, Masahiro Tsuchiya, Chiharu Ota, The Japan Environment and Children’s Study (JECS) Group, Michihiro Kamijima, Shin Yamazaki, Maki Fukami, Reiko Kishi, Chiharu Ota, Koichi Hashimoto, Chisato Mori, Shuichi Ito, Ryoji Shinohara, Hidekuni Inadera, Takeo Nakayama, Ryo Kawasaki, Yasuhiro Takeshima, Seiji Kageyama, Narufumi Suganuma, Shoichi Ohga, Takahiko Katoh

**Affiliations:** 1 Department of Feto-Maternal Medical Science, Tohoku University Graduate School of Medicine, 2-1, Seiryo-machi, Aoba-Ward, Sendai, Miyagi 980-8575 Japan; 2 Environment and Genome Research Center, Tohoku University Graduate School of Medicine, Sendai, Japan; 3 Department of Development and Environmental Medicine, Tohoku University Graduate School of Medicine, Sendai, Japan; 4 Department of Pediatrics, Tohoku University Hospital, Sendai, Japan; 5 Department of Nursing, Tohoku Fukushi University, Sendai, Japan

**Keywords:** Early childhood dental caries, Parental brushing, Extended breastfeeding, Parental smoking habit

## Abstract

**Background:**

Dental caries is estimated to affect almost half of preschool children worldwide and is associated with low academic performance in school age and poor health in adulthood. Thus, the prevention of dental caries is an important issue that must be addressed. The objective of this study was to clarify the factors associated with early childhood dental caries (ECC) in 4-year-old children.

**Methods:**

This study was part of an ongoing nationwide cohort study; the Japan Environment and Children’s Study. Approximately 100,000 expectant mothers were recruited from 15 Regional Centers between January 2011 and March 2014. Questionnaires were regularly administered to participating mothers. Data on the presence of ECC at 4 years of age, frequency of parental brushing, continuation of breastfeeding, parental smoking habits, and other factors considered to be related with development of ECC were obtained from the datasets released in October 2019 and April 2021. Then, the data were statistically analyzed.

**Results:**

After excluding 25,990 cases due to missing data referring to the prevalence of ECC, 74,310 cases were analyzed. The logistic regression analysis revealed that occasional parental brushing (adjusted odds ratio (aOR) 1.33, 95% confidence interval (95% CI) 1.27–1.39) was associated with significantly increased odds for ECC when compared to routine parental brushing. In addition, extended breastfeeding (aOR 2.07, 95% CI 1.86–2.29), continued smoking of mothers (aOR 1.42, 95% CI 1.30–1.55), and continued smoking of fathers (aOR 1.25, 95% CI 1.20–1.31) were associated with increased odds for ECC.

**Conclusion:**

Irregular parental brushing, extended breastfeeding, and parental smoking habits were found to be associated with increased odds for ECC in 4-year-old children.

**Supplementary Information:**

The online version contains supplementary material available at 10.1186/s12887-025-05997-8.

## Background

Dental caries is common among young children worldwide. It is one of the most common chronic diseases in children and its prevalence has been reported to be five times more common than asthma and seven times more common than hay fever [1]. It has been estimated that dental caries affects almost half of preschool children [2]. Early childhood dental caries (ECC) is defined as the presence of one or more decayed (noncavitated or cavitated lesions), missing (due to caries), or filled tooth surfaces in any primary tooth in a child under the age of 6 [3]. Although primary teeth are deciduous, children who have caries in these teeth are almost three times more likely to develop caries in their permanent teeth [4]. And dental caries in permanent teeth is associated with low academic performance [5]. Moreover, ECC is associated with poor health in adulthood [6]. Thus, preventing ECC is an important issue that must be addressed.

However, preventing ECC is difficult, as many factors have been reported to affect their development, and the effects of these factors remain open to debate in many cases. First, parental brushing has been reported to be associated with the prevention of ECC [7–9], while there is a study that demonstrated no association between them [10]. Second, some studies reported an association between extended breastfeeding and an increased likelihood of ECC [11], whereas others reported no significant association [12, 13]. In addition, there is limited data on the association between ECC prevalence and breastfeeding, extending up to higher ages, i.e., 3 or 4 years of age [14]. Third, while many studies have reported an association between parental smoking and an increased likelihood of developing ECC [15, 16], one study reported a stronger association between maternal smoking and ECC than that between paternal smoking and ECC [17], which is of interest for further evaluation. As ECC is a multifactorial disease, race, ethnicity, and customs of the target group should be considered to facilitate provision of effective countermeasures. Thus, a large cohort study is suitable for exploring the factors associated with the prevalence of ECC.

The Japan Environment and Children’s Study (JECS) is an ongoing nationwide government-funded birth cohort study. The goal of the JECS is to identify environmental factors that affect the health and development of children. Here, environmental factors include, not only chemical substances, such as lead or arsenic but also home environments, such as parental attitudes towards children and parental smoking habits. Based on the characteristics of the study design, the JECS is considered to be suitable for analyzing factors that are associated with the prevalence of ECC.

Thus, this study investigated the relationship between ECC and parental brushing frequency, extended breastfeeding, and parental smoking habits using data derived from the JECS.

## Methods

### Study design and setting

**Fig. 1:**
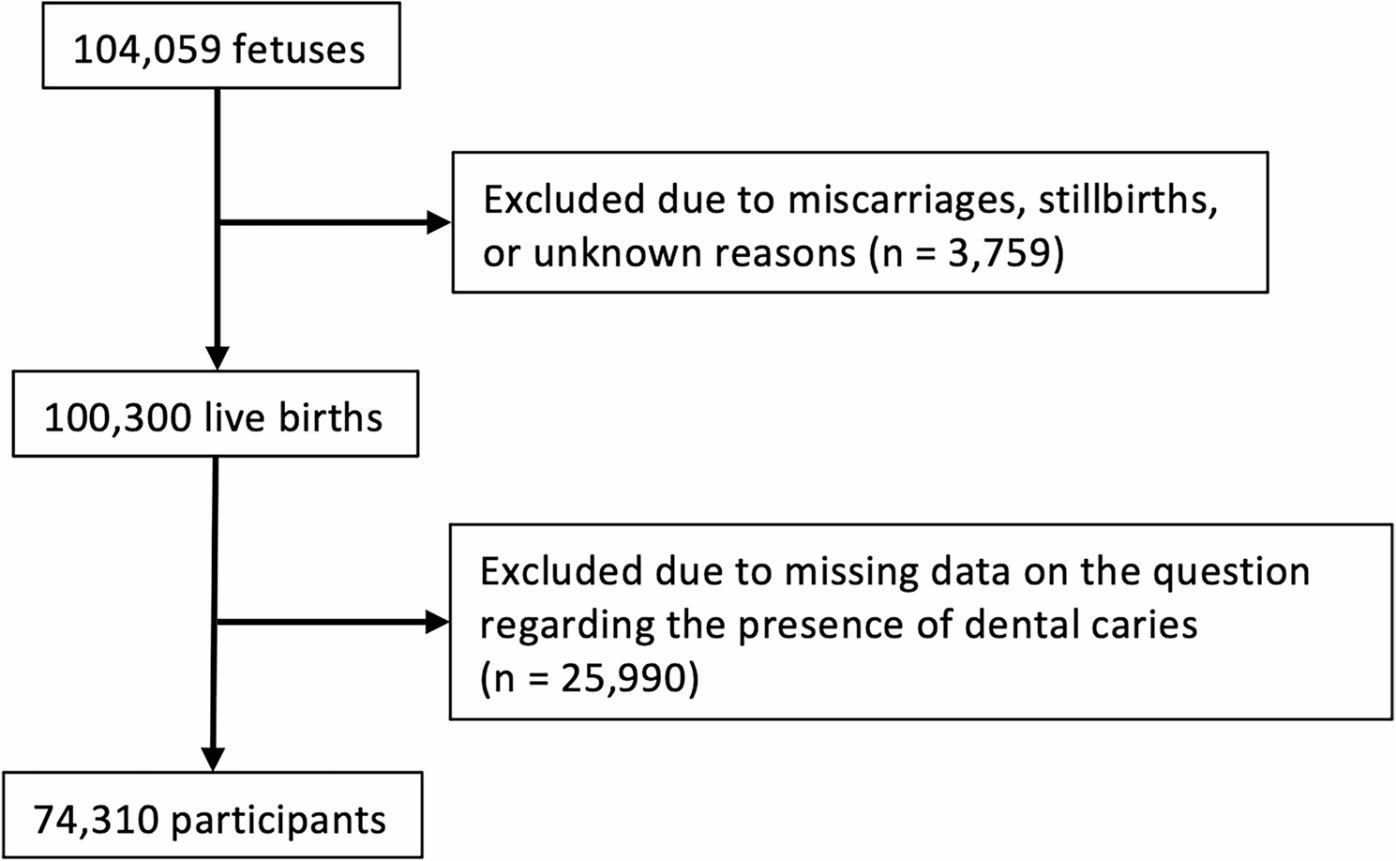
The flowchart of the study

This is part of an ongoing large Japanese cohort study, the JECS. The detailed methodology of the JECS has been described previously [18]. Approximately 100,000 expectant mothers were recruited from 15 Regional Centers (Hokkaido, Miyagi, Fukushima, Chiba, Kanagawa, Koshin, Toyama, Aichi, Kyoto, Osaka, Hyogo, Tottori, Kochi, Fukuoka, and South Kyusyu/Okinawa) between January 2011 and March 2014. Partners were also recruited, although participation was not mandatory. The JECS protocol was reviewed and approved by the Ministry of the Environment’s Institutional Review Board on Epidemiological Studies and by the Ethics Committees of all participating institutions. Written informed consent was obtained from the parents/guardians of all participating children, as well as from the participating parents themselves after they were informed of the aim and procedure of the study. A total of 104,059 fetal records were included, of which 100,300 were live births. After excluding 25,990 cases with missing data regarding the presence or absence of ECC at 4 years of age, 74,310 cases were analyzed (Fig. 1).

### Data acquisition

**Fig. 2:**
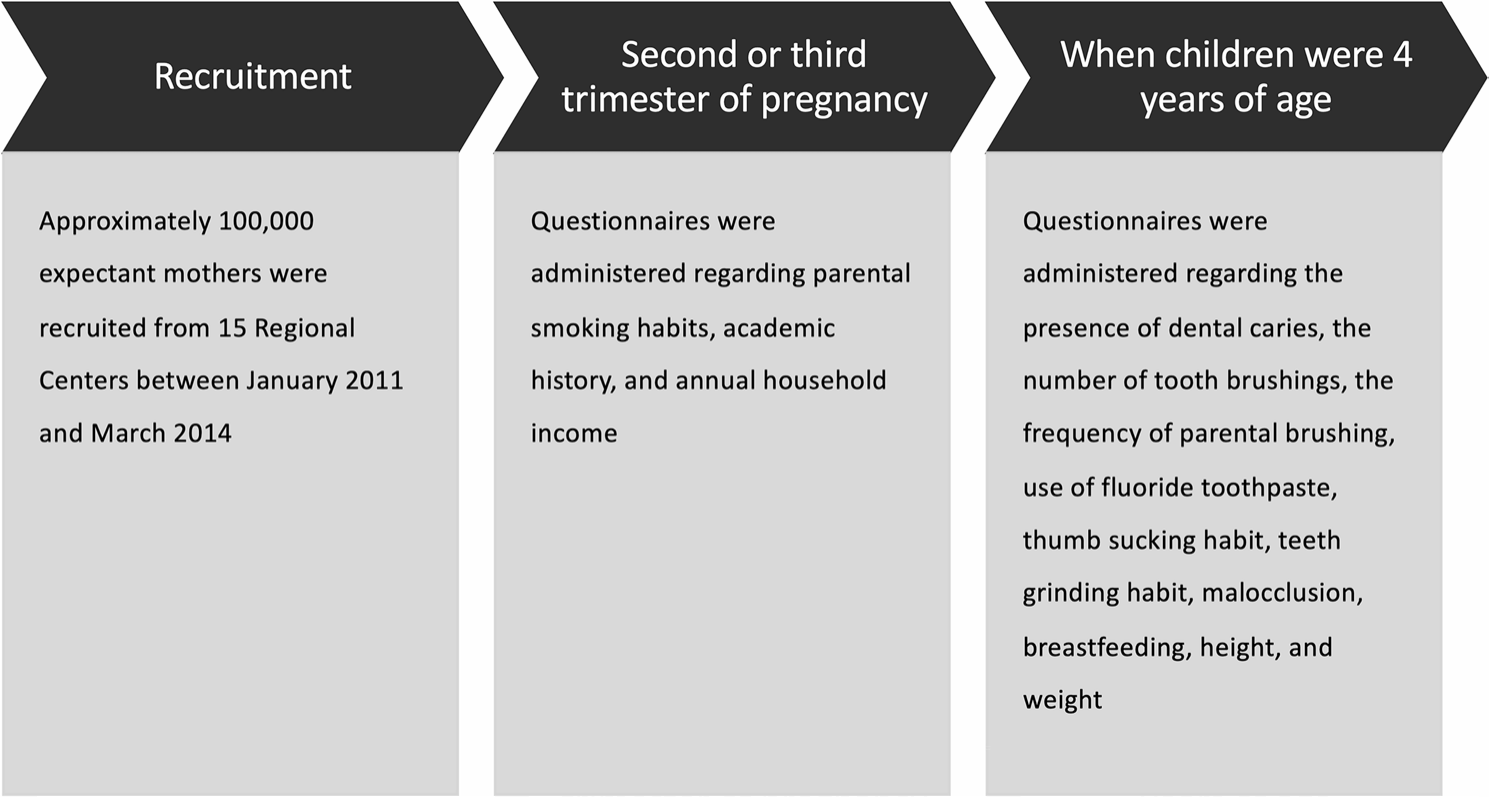
The timeline of the study

The timeline of this study is presented in Fig. 2. Questionnaires were administered to participating mothers, regularly, from after the first trimester of pregnancy. The jecs-ta-20190930-qsn and the jecs-qa-20210401-qsn data sets released in October 2019 and April 2021, respectively, were used. Data on the presence of ECC, frequency of parental brushing, number of brushings per day, use of fluoride toothpaste, thumb-sucking and bruxism habits, presence of malocclusion, continuation of breastfeeding, height, and weight were obtained from questionnaires administered to caregivers when their children were aged 4 years. And data on parental smoking habits, academic history, and annual household income were obtained from the questionnaires administered to mothers during the second or third trimester of pregnancy. Additionally, data on gestational weeks at birth were extracted from the medical record transcripts. The question-and-answer options are listed in Table 1. Regarding the continuation of breastfeeding, “previously breastfed; however, already ceased” and “exclusively formula-fed and never breastfed” were combined into one category. Similarly, the answers about parental smoking habits, “previously did, but quit before recognizing current pregnancy” and “previously did, but quit after finding out current pregnancy” were combined into one category.

**Table 1:** Questions and answer options administered to participants

Questions	Answer options
Has your child ever been diagnosed with dental caries by a dentist?	1. Yes 2. No
How many times does your child brush his or her teeth on a daily basis?	1. None 2. Once 3. Twice 4. Three times 5. More than four times
Do caregivers brush the teeth of the child?	1. Every time 2. Occasionally 3. Never
Does the toothpaste your child uses contain fluoride?	1. Yes 2. No
Does your child have a habit of thumb sucking?	1. Yes 2. No
Does your child have a habit of teeth grinding?	1. Yes 2. No
Has your child ever been diagnosed with malocclusion by a dentist?	1. Yes 2. No
Is your child still breastfeeding?	1. Previously breastfed; however, already ceased 2. Exclusively formula-fed and never breastfed 3. Yes, still breastfed
What is your highest education level?	1. Junior high school 2. High school 3. Technical college 4. Vocational school 5. Junior college 6. University 7. Graduate school
What is your partner’s highest education level?	1. Junior high school 2. High school 3. Technical college 4. Vocational school 5. Junior college 6. University 7. Graduate school
What is your smoking history?	1. Never smoked 2. Previously did, but quit before recognizing current pregnancy 3. Previously did, but quit after finding out current pregnancy 4. Still smoke
What is your partner’s smoking history?	1. Never smoked 2. Previously did, but quit before recognizing current pregnancy 3. Previously did, but quit after finding out current pregnancy 4. Still smoke
What is your household’s annual income?	1. Less than two million yen per year 2. Between two and four million yen per year 3. Between four and six million yen per year 4. Between six and eight million yen per year 5. Between eight and ten million yen per year 6. Between ten and twelve million yen per year 7. Between twelve and fifteen million yen per year 8. Between fifteen and twenty million yen per year 9. More than twenty million yen per year

### Data analysis

All statistical analyses were performed using R (version 4.5.0), and differences with *p* < 0.05 were considered statistically significant.

Missing values were evaluated using the R naniar package and were judged not to be missing completely at random. As Graham stated, all missing data should be considered a continuum between missing at random and missing not at random [19]. Thus, the data were treated as missing at random, and multiple imputations were performed to reduce the risk of potential bias. Multiple imputations were performed using the R mice package as follows: Missing values, except those regarding the presence or absence of ECC, were imputed multiple times by generating 20 independent datasets using chained equations. A fully conditional specification was used as the multiple imputation algorithm. After the imputation stage, logistic regression analyses were performed using a generalized linear model that included number of brushings per day, parental brushing frequency, use of fluoride toothpaste, thumb-sucking and bruxism habit, presence of malocclusion, continuation of breastfeeding, maternal and paternal academic history, maternal and paternal smoking habits, annual family income, child gender, gestational weeks, and child body mass index calculated with height and weight. Subsequently, adjusted odds ratios (aORs) and 95% confidence intervals (95% CIs) were calculated, and all results were combined based on Rubin’s rules. Multicollinearity was checked using the variance inflation factor for each explanatory variable. The Hosmer-Lemeshow test was performed to test the fit of the model.

For sensitivity analysis, the data was analyzed using three different methods. Sensitivity analysis 1: all cases with any missing data were excluded (*n* = 48,451; Additional Fig. 1). The data were fit to a generalized linear model, as described above; logistic regression analysis was performed, and aORs and 95% CIs were calculated. Sensitivity analysis 2: all the cases were included (*n* = 100,300; Additional Fig. 2), and all missing values were multiple imputed by generating 20 independent datasets using chained equations. After the imputation stage, logistic regression analyses were performed using the same model described above; aORs and 95% CIs were then calculated, and all results were combined based on Rubin’s rules. Sensitivity analysis 3: in addition to 25,990 cases with missing data regarding the presence or absence of ECC at 4 years of age, 1,655 cases with neurodevelopmental disorders or neoplastic diseases were excluded (*n* = 72,655; Additional Fig. 3). Then, missing values were multiple imputed by generating 20 independent datasets using chained equations. After the imputation stage, logistic regression analyses were performed using the same model described above; aORs and 95% CIs were then calculated, and all results were combined based on Rubin’s rules.

## Results

For the main analysis, the data from 74,310 participants were analyzed. The background of the participants is shown in Table 2. ECC was identified in 17,167 (23.1%) participants.

**Table 2:** Baseline characteristics of the participants

		Overall	Caries (-)	Caries (+)
*n*		74,310	57,143	17,167
Number of brushings (per day)	None	147 (0.2)	102 (0.2)	45 (0.3)
	1	17,942 (24.1)	13,646 (23.9)	4296 (25.0)
	2	35,660 (48.0)	27,229 (47.7)	8431 (49.1)
	3	20,251 (27.3)	15,917 (27.9)	4334 (25.2)
	> 4	271 (0.4)	222 (0.4)	49 (0.3)
	NA	39 (0.1)	27 (0.0)	12 (0.1)
Parental brushing	Every time	60,512 (81.4)	47,270 (82.7)	13,242 (77.1)
	Occasionally	12,493 (16.8)	8919 (15.6)	3574 (20.8)
	Never	474 (0.6)	343 (0.6)	131 (0.8)
	NA	831 (1.1)	611 (1.1)	220 (1.3)
Fluoride toothpaste	Not used	5057 (6.8)	3889 (6.8)	1168 (6.8)
	Used	55,999 (75.4)	43,050 (75.3)	12,949 (75.4)
	NA	13,254 (17.8)	10,204 (17.9)	3050 (17.8)
Thumb sucking habit	No	64,157 (86.3)	48,880 (85.5)	15,277 (89.0)
	Yes	10,071 (13.6)	8208 (14.4)	1863 (10.9)
	NA	82 (0.1)	55 (0.1)	27 (0.2)
Bruxism habit	No	57,347 (77.2)	44,087 (77.2)	13,260 (77.2)
	Yes	16,878 (22.7)	12,996 (22.7)	3882 (22.6)
	NA	85 (0.1)	60 (0.1)	25 (0.1)
Malocclusion	No	65,207 (87.7)	50,049 (87.6)	15,158 (88.3)
	Yes	8937 (12.0)	6979 (12.2)	1958 (11.4)
	NA	166 (0.2)	115 (0.2)	51 (0.3)
Breastfeeding	Never or ceased	70,859 (95.4)	54,743 (95.8)	16,116 (93.9)
	Still breastfed	1634 (2.2)	1025 (1.8)	609 (3.5)
	NA	1817 (2.4)	1375 (2.4)	442 (2.6)
Maternal education	Junior high school	2554 (3.4)	1658 (2.9)	896 (5.2)
	High school	21,535 (29.0)	15,515 (27.2)	6020 (35.1)
	Technical college	1159 (1.6)	897 (1.6)	262 (1.5)
	Vocational school	17,095 (23.0)	13,311 (23.3)	3784 (22.0)
	Junior college	13,597 (18.3)	10,728 (18.8)	2869 (16.7)
	University	16,367 (22.0)	13,417 (23.5)	2950 (17.2)
	Graduate school	1202 (1.6)	1004 (1.8)	198 (1.2)
	NA	801 (1.1)	613 (1.1)	188 (1.1)
Paternal education	Junior high school	4499 (6.1)	2984 (5.2)	1515 (8.8)
	High school	25,619 (34.5)	18,820 (32.9)	6799 (39.6)
	Technical college	1580 (2.1)	1243 (2.2)	337 (2.0)
	Vocational school	13,710 (18.4)	10,654 (18.6)	3056 (17.8)
	Junior college	1559 (2.1)	1237 (2.2)	322 (1.9)
	University	22,500 (30.3)	18,221 (31.9)	4279 (24.9)
	Graduate school	3688 (5.0)	3132 (5.5)	556 (3.2)
	NA	1155 (1.6)	852 (1.5)	303 (1.8)
Maternal smoking habit	Never smoked	44,616 (60.0)	35,340 (61.8)	9276 (54.0)
	Smoked, but quit	26,266 (35.3)	19,529 (34.2)	6737 (39.2)
	Still smoke	2471 (3.3)	1557 (2.7)	914 (5.3)
	NA	957 (1.3)	717 (1.3)	240 (1.4)
Paternal smoking habit	Never smoked	21,233 (28.6)	17,160 (30.0)	4073 (23.7)
	Smoked, but quit	19,869 (26.7)	15,856 (27.7)	4013 (23.4)
	Still smoke	31,661 (42.6)	22,994 (40.2)	8667 (50.5)
	NA	1547 (2.1)	1133 (2.0)	414 (2.4)
Family income (million yen per year)	< 2	3309 (4.5)	2218 (3.9)	1091 (6.4)
	2–4	23,071 (31.0)	17,248 (30.2)	5823 (33.9)
	4–6	23,349 (31.4)	18,212 (31.9)	5137 (29.9)
	6–8	11,594 (15.6)	9345 (16.4)	2249 (13.1)
	8–10	4780 (6.4)	3814 (6.7)	966 (5.6)
	10–12	1793 (2.4)	1448 (2.5)	345 (2.0)
	12–15	687 (0.9)	543 (1.0)	144 (0.8)
	15–20	410 (0.6)	314 (0.5)	96 (0.6)
	> 20	230 (0.3)	174 (0.3)	56 (0.3)
	NA	5087 (6.8)	3827 (6.7)	1260 (7.3)
Gender	Male	38,027 (51.2)	28,879 (50.5)	9148 (53.3)
	Female	36,283 (48.8)	28,264 (49.5)	8019 (46.7)
Gestational weeks (weeks)		39.0 [2.0]	39.0 [2.0]	39.0 [2.0]
Body mass index (kg•m^−2^)		15.6 [1.4]	15.6 [1.4]	15.6 [1.4]

All variables except for gestational weeks and body mass index are expressed as number (%). Gestational weeks and body mass index are expressed as median [interquartile range]

*NA* No answer

The odds of ECC were 0.28 (13,242/47,270) in children who always received assistance with teeth brushing from caregivers, 0.40 (3,574/8,919) in those who occasionally received assistance with brushing from their caregivers, and 0.38 (131/343) in those who never received assistance with brushing from their caregivers. The logistic regression analysis revealed that occasional parental brushing was associated with a significantly increased aOR of their children having ECC (aOR 1.33, 95% CI 1.27–1.39, Table 3). Meanwhile, children who never received assistance with brushing from their parents were not associated with a significantly increased aOR for ECC (aOR 1.12, 95% CI 0.91–1.37, Table 3). Extended breastfeeding was associated with a significantly increased aOR for ECC (aOR 2.07, 95% CI 1.86–2.29, Table 3). Furthermore, the continued smoking habit of mothers (aOR 1.42, 95% CI 1.30–1.55) and that of fathers (aOR 1.25, 95% CI 1.20–1.31) were associated with significantly increased aORs of their children having ECC as well (Table 3). Notably, even if mothers quit smoking before the birth of their children, the aOR remained higher when compared with children whose mothers had no smoking experience (aOR 1.10, 95% CI 1.06–1.14, Table 3). Additionally, female gender was associated with decreased aOR for ECC (aOR 0.89, 95% CI 0.86–0.92).

**Table 3:** Crude and adjusted odds ratios computed after multiple imputation (*n* = 74,310)

	Crude odds ratio (95% CI)	Adjusted odds ratio (95% CI)
Parental brushing		
Every time (reference)	1	1
Occasionally	1.43 (1.37–1.50)	1.33 (1.27–1.39)
Never	1.40 (1.15–1.71)	1.12 (0.91–1.37)
Breastfeeding		
Never or ceased (reference)	1	1
Still breastfed	2.02 (1.83–2.24)	2.07 (1.86–2.29)
Maternal smoking habit		
Never (reference)	1	1
Quit	1.32 (1.27–1.36)	1.10 (1.06–1.14)
Still smoke	2.23 (2.05–2.43)	1.42 (1.30–1.55)
Paternal smoking habit		
Never (reference)	1	1
Quit	1.07 (1.02–1.12)	0.98 (0.93–1.03)
Still smoke	1.59 (1.53–1.66)	1.25 (1.20–1.31)
Gender		
Boy (reference)	1	1
Girl	0.90 (0.87–0.93)	0.89 (0.86–0.92)

Adjusted odds ratios were calculated using a generalized linear model that included number of brushings per day, parental brushing frequency, use of fluoride toothpaste, thumb-sucking and bruxism habit, presence of malocclusion, continuation of breastfeeding, maternal and paternal academic history, maternal and paternal smoking habits, annual family income, child gender, gestational weeks, and child body mass index calculated with height and weight

*95% CI* 95% confidence interval

The logistic regression analysis using data that excluded all missing values (Additional Table 1), that using all data (Additional Table 2), or that using data excluding cases with neurodevelopmental disorders or neoplastic diseases (Additional Table 3) exhibited no significant differences compared to the results shown in Table 3 (Tables 4, 5 and 6).

**Table 4:** Crude and adjusted odds ratios computed after excluding all missing values (*n* = 48,451)

	Crude odds ratio (95% CI)	Adjusted odds ratio (95% CI)
Parental brushing		
Every time (reference)	1	1
Occasionally	1.40 (1.33–1.48)	1.31 (1.23–1.38)
Never	1.20 (0.89–1.61)	0.97 (0.71–1.30)
Breastfeeding		
Never or ceased (reference)	1	1
Still breastfed	2.09 (1.84–2.38)	2.12 (1.86–2.41)
Maternal smoking habit		
Never (reference)	1	1
Quit	1.31 (1.26–1.37)	1.11 (1.06–1.16)
Still smoke	2.20 (1.98–2.46)	1.44 (1.28–1.61)
Paternal smoking habit		
Never (reference)	1	1
Quit	1.10 (1.04–1.17)	1.01 (0.95–1.07)
Still smoke	1.59 (1.51–1.68)	1.27 (1.20–1.34)
Gender		
Boy (reference)	1	1
Girl	0.89 (0.86–0.93)	0.89 (0.85–0.93)

Adjusted odds ratios were calculated using a generalized linear model that included number of brushings per day, parental brushing frequency, use of fluoride toothpaste, thumb-sucking and bruxism habit, presence of malocclusion, continuation of breastfeeding, maternal and paternal academic history, maternal and paternal smoking habits, annual family income, child gender, gestational weeks, and child body mass index calculated with height and weight

*95% CI* 95% confidence interval

**Table 5:** Crude and adjusted odds ratios computed after multiple imputation with all data (*n* = 100,300)

	Crude odds ratio (95% CI)	Adjusted odds ratio (95% CI)
Parental brushing		
Every time (reference)	1	1
Occasionally	1.45 (1.39–1.51)	1.32 (1.27–1.38)
Never	1.48 (1.22–1.78)	1.21 (0.99–1.46)
Breastfeeding		
Never or ceased (reference)	1	1
Still breastfed	2.01 (1.81–2.22)	2.05 (1.85–2.28)
Maternal smoking habit		
Never (reference)	1	1
Quit	1.33 (1.28–1.38)	1.10 (1.06–1.14)
Still smoke	2.24 (2.05–2.44)	1.41 (1.28–1.55)
Paternal smoking habit		
Never (reference)	1	1
Quit	1.07 (1.02–1.13)	0.98 (0.94–1.03)
Still smoke	1.62 (1.55–1.69)	1.26 (1.20–1.32)
Gender		
Boy (reference)	1	1
Girl	0.90 (0.87–0.93)	0.89 (0.86–0.93)

Adjusted odds ratios were calculated using a generalized linear model that included number of brushings per day, parental brushing frequency, use of fluoride toothpaste, thumb-sucking and bruxism habit, presence of malocclusion, continuation of breastfeeding, maternal and paternal academic history, maternal and paternal smoking habits, annual family income, child gender, gestational weeks, and child body mass index calculated with height and weight

*95% CI* 95% confidence interval

**Table 6:** Crude and adjusted odds ratios computed after multiple imputation on data excluding cases with neurodevelopmental disorders or neoplastic diseases (*n* = 72,655)

	Crude odds ratio (95% CI)	Adjusted odds ratio (95% CI)
Parental brushing		
Every time (reference)	1	1
Occasionally	1.43 (1.37–1.50)	1.33 (1.27–1.39)
Never	1.35 (1.10–1.66)	1.08 (0.87–1.33)
Breastfeeding		
Never or ceased (reference)	1	1
Still breastfed	1.99 (1.80–2.21)	2.04 (1.83–2.26)
Maternal smoking habit		
Never (reference)	1	1
Quit	1.32 (1.27–1.37)	1.10 (1.06–1.15)
Still smoke	2.23 (2.05–2.43)	1.42 (1.30–1.56)
Paternal smoking habit		
Never (reference)	1	1
Quit	1.07 (1.02–1.12)	0.98 (0.93–1.03)
Still smoke	1.59 (1.53–1.66)	1.25 (1.20–1.31)
Gender		
Boy (reference)	1	1
Girl	0.89 (0.86–0.92)	0.89 (0.86–0.92)

Adjusted odds ratios were calculated using a generalized linear model that included number of brushings per day, parental brushing frequency, use of fluoride toothpaste, thumb-sucking and bruxism habit, presence of malocclusion, continuation of breastfeeding, maternal and paternal academic history, maternal and paternal smoking habits, annual family income, child gender, gestational weeks, and child body mass index calculated with height and weight

*95% CI* 95% confidence interval

## Discussion

In this study, using a large birth cohort in which ECC was observed in approximately 23% of children at 4 years of age, several factors were shown to be related to the odds of ECC.

First, if parents did not brush their children’s teeth each time, the odds of ECC increased (Tables 3, 4, 5 and 6). Although it must be mentioned that the aORs in the “never” group in parental brushing were not statistically significant, these results should be considered with caution. It is illogical that there was no association between no parental brushing and increased aOR for ECC, whereas occasional parental brushing was associated with increased aOR for ECC. Using pwr.2p2n.test, the needed sample size was calculated as 457.7, when the statistical power and the significance level were defined as 0.8 and 0.05, respectively. The actual sample number of the “never” group was 474 (Table 2), and this was only marginally above the needed sample size. This might explain why statistical significance was not detected after adjustment with multiple covariates. As this was a cross-sectional study, a causal relationship could not be established; therefore, this relationship is discussed based on the findings of previous studies. Dental caries develops when bacteria in the plaque on tooth surfaces ferment carbohydrates, especially sucrose and glucose, and produce acids, resulting in tooth enamel dissolution. These bacteria include *Streptococcus mutans*, *Actinomyces*, *Lactobacillus*, *Bifidobacterium*, and *Candida albicans* [20]. Among these, *S. mutans* plays a major role in providing a matrix of biofilms and protecting the acidic environment in which other acidogenic and aciduric organisms thrive [21]. Tooth brushing prevents development of dental caries by mechanically removing plaque [20]. Our results are consistent with those of previous studies [8, 9, 22], and fit with the assumption that children less than 4 years of age may not have the proficiency to brush their teeth thoroughly by themselves. More than 17% of parents (12,967/74,310; Table 2) did not brush their children’s teeth every time, probably because they were too busy [23] or because they believed that their children were old enough to proficiently brush their teeth. However, Deinzer et al. reported that more than half of children at 12 years of age could not proficiently brush their teeth [24], and Sandström et al. reported that children at 6 years of age brushed their teeth less proficiently than children at 10 years of age [25]. Considering that children acquire better brushing technique as they grow, children at 4 years of age may be even less proficient at brushing their teeth than children at 6 years of age. If half of children at age 12 were unable to brush their teeth proficiently, as reported by Deinzer, a much higher proportion of children at age 4 would fail to brush their teeth proficiently. Thus, the teeth of 4-year-old children should be brushed by their parents/caregivers. In addition, based on the results of this study, parental brushing every time, not occasionally, may be necessary to prevent ECC. Therefore, an educational campaign highlighting the importance of routine parental brushing is warranted.

Second, extended breastfeeding up to age 4 was associated with 107% increased odds of a child having ECC (Table 3). This finding is consistent with those of the previous studies [7, 11, 26]. However, the exact cause of this has not yet been elucidated. Among children who breastfeed for an extended period, nocturnal feeding is another risk factor for ECC. Tham et al. stated that the tooth surfaces of infants are exposed to breast milk for a prolonged period after nocturnal breastfeeding, which may increase the risk of ECC development [27]. Because there were no questions regarding nocturnal feeding, information on this topic could not be collected in this study. On the other hand, breast milk has been reported to have low cariogenic potential [28, 29], and a protective effect of breast milk against ECC was reported in an in vitro study [30]. In addition, breastfeeding is associated with higher intelligence and protective effects against infectious diseases [31]. In this study, 2.2% of children at age 4 were still breastfed. It is possible that their mothers continued breastfeeding to avoid undernutrition when the nutritional transition was insufficient, although the reason why breastfeeding was continued was not collected in this study. Thus, the results of the present study do not encourage mothers to quit breastfeeding. Further studies are required to explore the causal relationship between extended breastfeeding and ECC.

Third, parental smoking habits related to the increased odds of their children having ECC at age 4. Maternal smoking habits exhibited higher aORs than those with paternal smoking habits (Tables 3, 4, 5 and 6), which is in line with the results of a previous study that reported a stronger association between maternal smoking habits and ECC than between paternal smoking habits and ECC [17]. Leroy et al. reported a significant relationship between parental smoking habits and ECC in children aged 5 years [15]. Children would be exposed to passive smoking when their parents smoke and several studies have confirmed the relationship between passive smoking and ECC [16, 32, 33]. Nicotine in tobacco promotes the growth of cariogenic microorganisms, including *Streptococcus mutans* and *Lactobacilli*. These bacteria create the preconditions for dental caries by producing acid [34]. In addition to promoting the growth of cariogenic microorganisms through passive nicotine inhalation, sharing of utensils between mother and child leads to transmission of bacteria-rich saliva [35]. If the mother has cariogenic microorganisms due to her smoking habit, those microorganisms are transferred to the child, which results in the development of ECC. This may explain the reason for the stronger association between maternal smoking habits and ECC. Interestingly, maternal smoking habits affected the children even after the mothers quit smoking (Tables 3, 4, 5 and 6), suggesting that cariogenic bacterial flora remained unchanged. While some studies using nasopharyngeal samples [36] and subgingival plaque [37] have reported that the bacterial flora of smokers changed after cessation of smoking. These results suggest that although the cessation of smoking was not early enough in this study, if mothers ceased smoking earlier, their children might not be at a high risk of developing ECC. Thus, it is important to inform women of reproductive age before pregnancy about the risks of smoking-related ECC in their forthcoming children. It is vital to encourage individuals to cease smoking if they currently smoke.

Additionally, female gender was associated with 11% of decreased odds of a child having ECC (Tables 3, 4, 5 and 6). Previous studies have demonstrated mixed results regarding the association between child gender and ECC. Some studies reported no significant relationship between them [9, 11, 38], while a study conducted in Japan reported decreased odds of ECC in female [39]. It may be related to genetic factors; thus, further studies are warranted to investigate the relationship between child gender and ECC.

To extrapolate the results of this study, it is important to compare them to those of previous cohort studies conducted in other countries. A birth cohort study with 1,429 participants conducted in the UK reported higher hazard odds of the first occurrence of ECC in children whose parents smoked than in children whose parents did not smoke [40]. Another birth cohort study conducted in Brazil uncovered an association between breastfeeding for longer than 24 months and an elevated prevalence of severe ECC compared to breastfeeding for 6–23 months [41]. Moreover, a German birth cohort study discovered an association between supervised/regular second brushing by parents and a low odds ratio of ECC experience in 5-year-old children [42]. The findings of this study are consistent with those of previous studies; thus, our findings can be applied to children around the world.

This study had some limitations that should be noted when interpreting our findings. First, it was a retrospective analysis using data that were not necessarily aimed at studying ECC. Therefore, the questionnaires were not a perfect fit for the analysis; namely, in addition to the lack of a question regarding nocturnal feeding described above, there were no questions regarding the number of dental caries, eating habit including frequency of sweet food, fruit, and vegetable intake [43] at age 4, or parental history of dental caries. Furthermore, the questionnaires were answered by caregivers, and the data lacked a direct assessment by dentists. Thus, it was not possible to assess the dmft (decayed, missing, and filled teeth) score, which is a key measure of ECC. Moreover, there may be recall or reporting bias owing to the characteristics of the study design. Indeed, 25,990 participants did not answer the question regarding the presence or absence of ECC, suggesting a potential reporting bias. However, previous studies conducted around a similar period in Japan reported the prevalence of ECC as 14.7–20.6% at age 3 [16, 44, 45] and 44.4% at age 6 or 7 [46]. Thus, the prevalence of ECC at age 4 was hypothesized to be between 14.7% and 44.4%. Assuming a linear relationship, the prevalence of ECC at age 4 is calculated to be around 22–28%, which do not deviate substantially from the results of this study. As such, the prevalence of ECC reported in this study seems reasonable, thus suggesting that the potential biases might not be too large. Finally, although several other factors have been reported to be related to ECC, such as sleep duration [47], maternal vitamin D level [48], child vitamin D level [49], problematic screen exposure [50], and child temperament [51], these factors were not investigated in this study. Therefore, future studies including these factors are warranted.

On the other hand, this was a nationwide survey, and the sample was representative of the general Japanese population. Thus, the strength of this study was its large sample size. Additionally, the analyses were replicated using four datasets, including those in which missing data were imputed multiple times, to reduce biases when possible.

## Conclusions

In conclusion, irregular parental brushing was associated with an increased odds of ECC in 4-year-old children. Extended breastfeeding and parental smoking habits were also associated with an increased odds of ECC. In addition, maternal smoking habit had a stronger association with ECC than paternal smoking habit. Further studies are needed to investigate the causal effects of these factors on ECC.

## Supplementary Information

Supplementary Material 1.

Supplementary Material 2.

Supplementary Material 3.

Supplementary Material 4.

## Data Availability

Data are unsuitable for public deposition due to ethical restrictions and legal framework of Japan. It is prohibited by the Act on the Protection of Personal Information (Act No. 57 of 30 May 2003, amendment on 9 September 2015) to publicly deposit the data containing personal information. Ethical Guidelines for Medical and Health Research Involving Human Subjects enforced by the Japan Ministry of Education, Culture, Sports, Science and Technology and the Ministry of Health, Labour and Welfare also restricts the open sharing of the epidemiologic data. All inquiries about access to data should be sent to: jecs-en@nies.go.jp. The person responsible for handling enquiries sent to this e-mail address is Dr Shoji F. Nakayama, JECS Programme Office, National Institute for Environmental Studies.

## Abbreviations

ECC: Early childhood dental caries
JECS: The Japan Environment and Children’s Study
aOR: Adjusted odds ratio
95% CI: 95% confidence interval
dmft scores: Decayed, missing, and filled teeth scores
