# Supplementary figures and images for: A cross-sectional study investigating the association between parental daily brushing, extended breastfeeding, or parental smoking habit and early childhood dental caries in 4-year-old children: the Japan Environment and Children’s Study
Source: BMC Pediatr. 2025 Aug 8;25:613. doi: 10.1186/s12887-025-05997-8 (PMC12333281; doi:10.1186/s12887-025-05997-8)

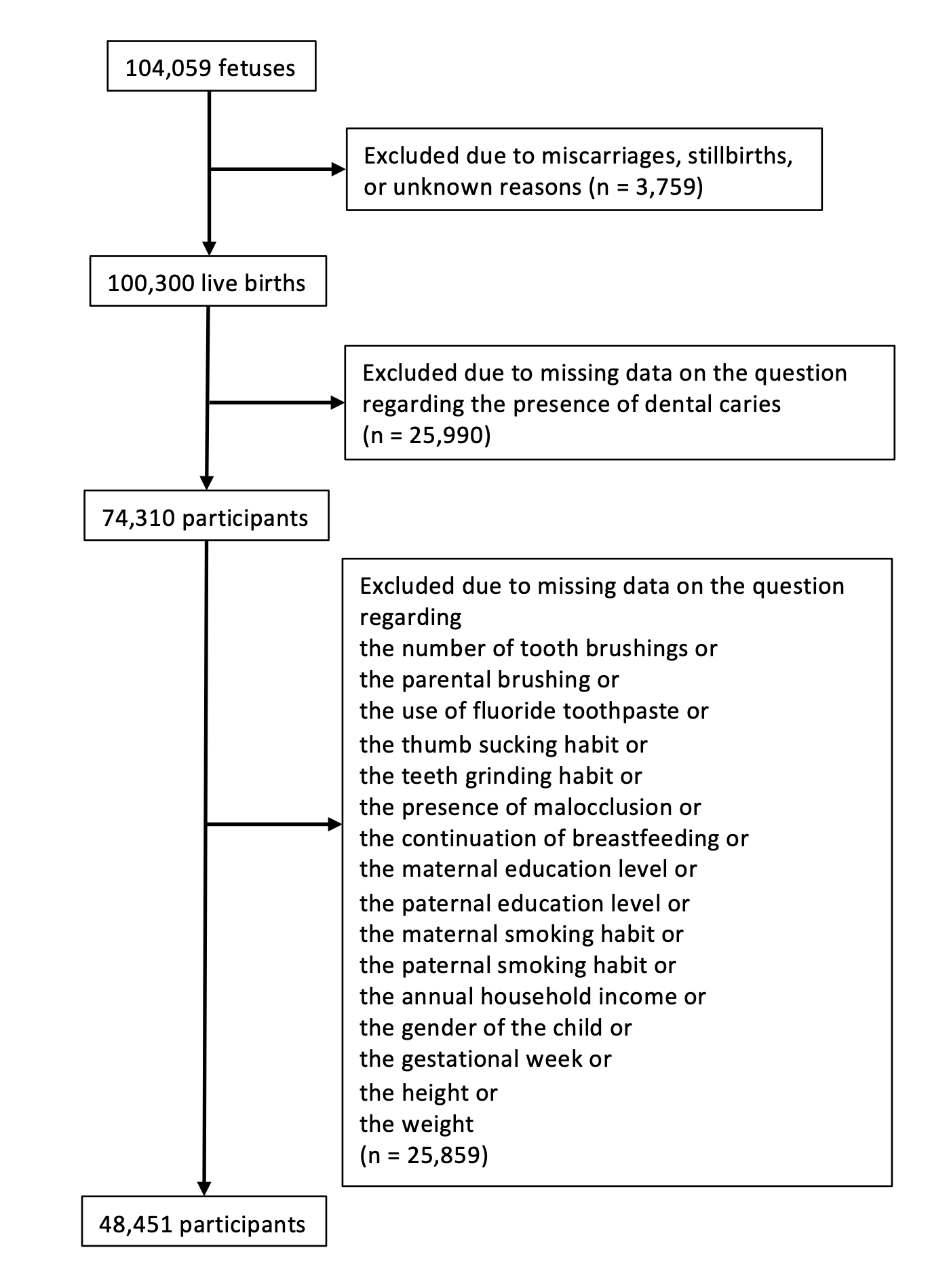

Supplement: Supplementary file 1 — Supplementary Material 1. [file 12887_2025_5997_MOESM1_ESM.tiff]

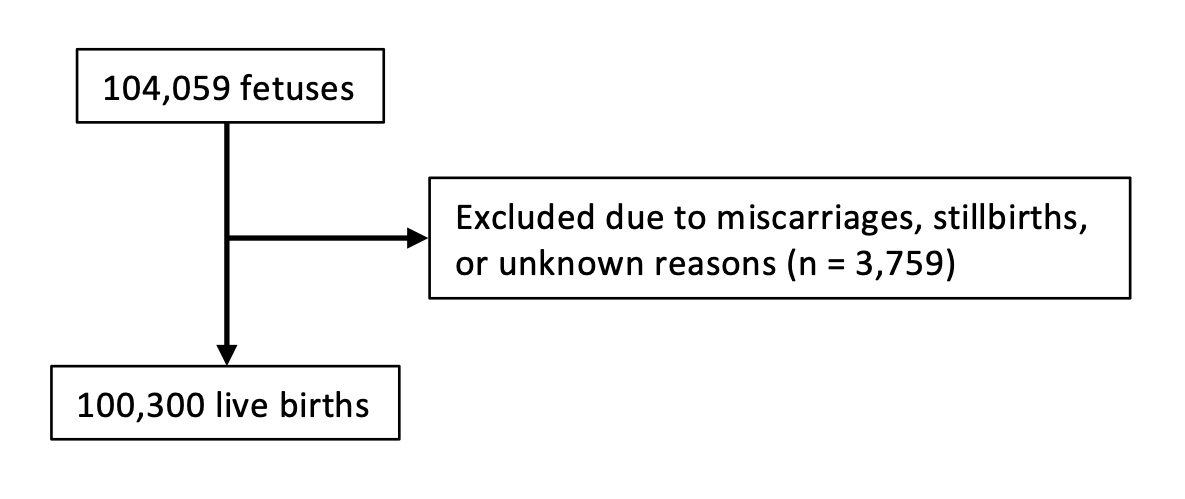

Supplement: Supplementary file 2 — Supplementary Material 2. [file 12887_2025_5997_MOESM2_ESM.tiff]

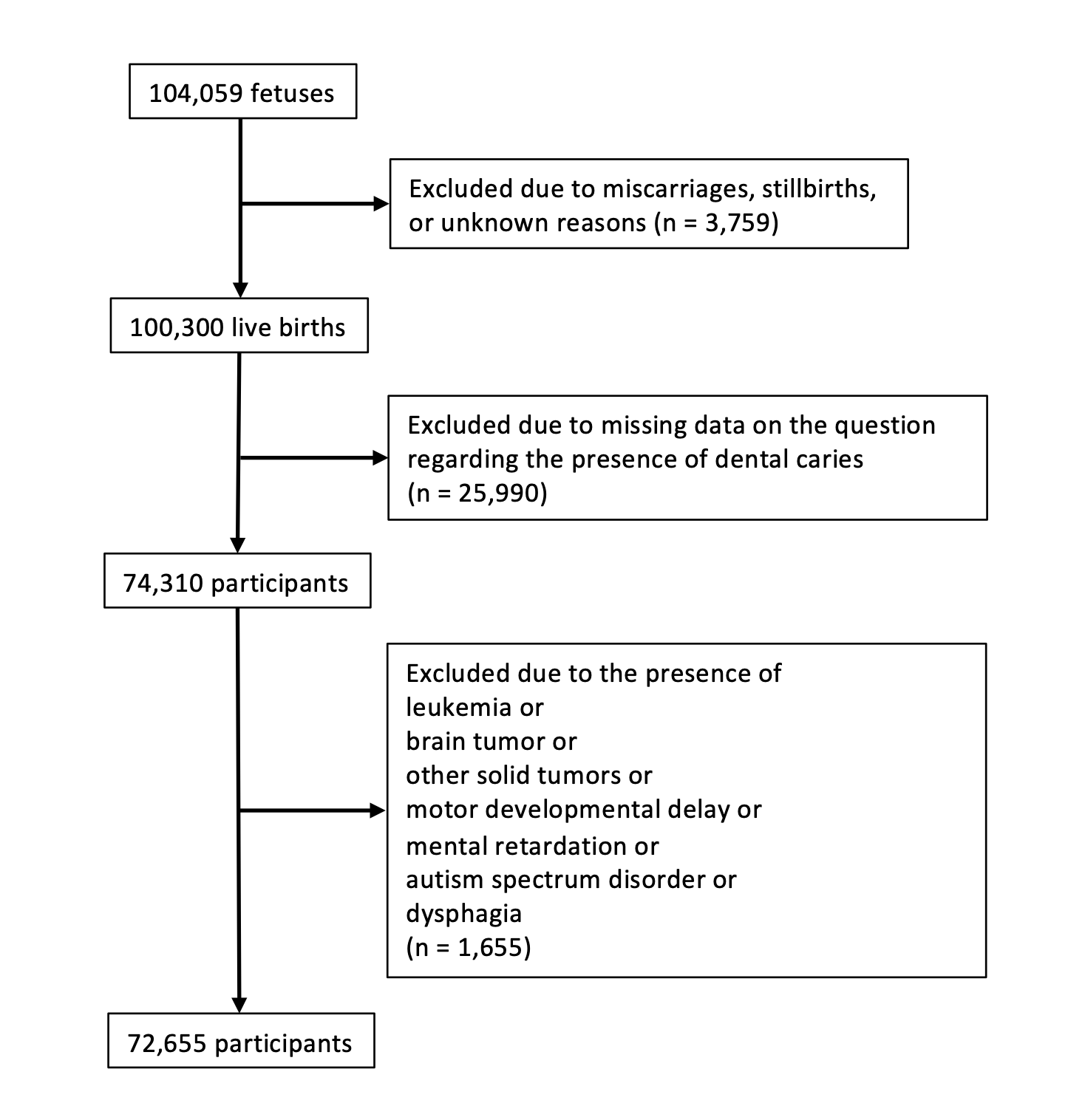

Supplement: Supplementary file 3 — Supplementary Material 3. [file 12887_2025_5997_MOESM3_ESM.tiff]
