# Supplementary material for: A cross-sectional study investigating the association between parental daily brushing, extended breastfeeding, or parental smoking habit and early childhood dental caries in 4-year-old children: the Japan Environment and Children’s Study
Source: BMC Pediatr. 2025 Aug 8;25:613. doi: 10.1186/s12887-025-05997-8 (PMC12333281; doi:10.1186/s12887-025-05997-8)
Supplement: Supplementary file 4 — Supplementary Material 4. [file 12887_2025_5997_MOESM4_ESM.docx]

Additional Table 1. Baseline characteristics of the participants after excluding all missing values (n = 48,451).

|  |  | Overall | Caries (-) | Caries (+) |
| --- | --- | --- | --- | --- |
| n |  | 48451 | 37563 | 10888 |
| Number of  brushings  (per day) | None | 11 (0.0) | 9 (0.0) | 2 (0.0) |
|  | 1 | 10803 (22.3) | 8277 (22.0) | 2526 (23.2) |
|  | 2 | 23508 (48.5) | 18060 (48.1) | 5448 (50.0) |
|  | 3 | 13958 (28.8) | 11080 (29.5) | 2878 (26.4) |
|  | >4 | 171 (0.4) | 137 (0.4) | 34 (0.3) |
| Parental  brushing | Every time | 40546 (83.7) | 31842 (84.8) | 8704 (79.9) |
|  | Occasionally | 7666 (15.8) | 5541 (14.8) | 2125 (19.5) |
|  | Never | 239 (0.5) | 180 (0.5) | 59 (0.5) |
| Fluoride  toothpaste | Not used | 3944 (8.1) | 3058 (8.1) | 886 (8.1) |
|  | Used | 44507 (91.9) | 34505 (91.9) | 10002 (91.9) |
| Thumb  sucking habit | No | 41979 (86.6) | 32259 (85.9) | 9720 (89.3) |
|  | Yes | 6472 (13.4) | 5304 (14.1) | 1168 (10.7) |
| Teeth  grinding habit | No | 37337 (77.1) | 28921 (77.0) | 8416 (77.3) |
|  | Yes | 11114 (22.9) | 8642 (23.0) | 2472 (22.7) |
| Malocclusion | No | 42687 (88.1) | 33019 (87.9) | 9668 (88.8) |
|  | Yes | 5764 (11.9) | 4544 (12.1) | 1220 (11.2) |
| Breastfeeding | Never or ceased | 47403 (97.8) | 36906 (98.3) | 10497 (96.4) |
|  | Still breastfed | 1048 (2.2) | 657 (1.7) | 391 (3.6) |
| Maternal education | Junior high school | 1457 (3.0) | 970 (2.6) | 487 (4.5) |
|  | High school | 13475 (27.8) | 9785 (26.0) | 3690 (33.9) |
|  | Technical college | 802 (1.7) | 621 (1.7) | 181 (1.7) |
|  | Vocational school | 11469 (23.7) | 8995 (23.9) | 2474 (22.7) |
|  | Junior college | 9212 (19.0) | 7279 (19.4) | 1933 (17.8) |
|  | University | 11209 (23.1) | 9231 (24.6) | 1978 (18.2) |
|  | Graduate school | 827 (1.7) | 682 (1.8) | 145 (1.3) |
| Paternal education | Junior high school | 2732 (5.6) | 1838 (4.9) | 894 (8.2) |
|  | High school | 16673 (34.4) | 12322 (32.8) | 4351 (40.0) |
|  | Technical college | 1051 (2.2) | 835 (2.2) | 216 (2.0) |
|  | Vocational school | 9108 (18.8) | 7134 (19.0) | 1974 (18.1) |
|  | Junior college | 1012 (2.1) | 816 (2.2) | 196 (1.8) |
|  | University | 15328 (31.6) | 12450 (33.1) | 2878 (26.4) |
|  | Graduate school | 2547 (5.3) | 2168 (5.8) | 379 (3.5) |
| Maternal smoking habit | Never smoked | 29603 (61.1) | 23601 (62.8) | 6002 (55.1) |
|  | Smoked, but quit | 17333 (35.8) | 12991 (34.6) | 4342 (39.9) |
|  | Still smoke | 1515 (3.1) | 971 (2.6) | 544 (5.0) |
| Paternal smoking habit | Never smoked | 14247 (29.4) | 11596 (30.9) | 2651 (24.3) |
|  | Smoked, but quit | 13548 (28.0) | 10825 (28.8) | 2723 (25.0) |
|  | Still smoke | 20656 (42.6) | 15142 (40.3) | 5514 (50.6) |
| Family income | <2 | 2075 (4.3) | 1395 (3.7) | 680 (6.2) |
| (million yen per year) | 2-4 | 15923 (32.9) | 12019 (32.0) | 3904 (35.9) |
|  | 4-6 | 16555 (34.2) | 12944 (34.5) | 3611 (33.2) |
|  | 6-8 | 8283 (17.1) | 6718 (17.9) | 1565 (14.4) |
|  | 8-10 | 3431 (7.1) | 2760 (7.3) | 671 (6.2) |
|  | 10-12 | 1278 (2.6) | 1028 (2.7) | 250 (2.3) |
|  | 12-15 | 474 (1.0) | 366 (1.0) | 108 (1.0) |
|  | 15-20 | 276 (0.6) | 213 (0.6) | 63 (0.6) |
|  | >20 | 156 (0.3) | 120 (0.3) | 36 (0.3) |
| Gender | Male | 24782 (51.1) | 18977 (50.5) | 5805 (53.3) |
|  | Female | 23669 (48.9) | 18586 (49.5) | 5083 (46.7) |
| Gestational weeks (weeks) |  | 39.0 [2.0] | 39.0 [2.0] | 39.0 [2.0] |
| Body mass index (kg•m^-2^) |  | 15.6 [1.4] | 15.6 [1.4] | 15.6 [1.5] |

All variables except for gestational weeks and body mass index are expressed as number (%). Gestational weeks and body mass index are expressed as median [interquartile range].

Additional Table 2. Baseline characteristics of all the participants (n = 100,300).

|  |  | Overall | Caries (-) | Caries (+) |
| --- | --- | --- | --- | --- |
| n |  | 100300 | 57143 | 17167 |
| Number of  brushings  (per day) | None | 159 (0.2) | 102 (0.2) | 45 (0.3) |
|  | 1 | 18758 (18.7) | 13646 (23.9) | 4296 (25.0) |
|  | 2 | 37671 (37.6) | 27229 (47.7) | 8431 (49.1) |
|  | 3 | 21193 (21.1) | 15917 (27.9) | 4334 (25.2) |
|  | >4 | 282 (0.3) | 222 (0.4) | 49 (0.3) |
|  | NA | 22237 (22.2) | 27 (0.0) | 12 (0.1) |
| Parental  brushing | Every time | 63338 (63.1) | 47270 (82.7) | 13242 (77.1) |
|  | Occasionally | 13379 (13.3) | 8919 (15.6) | 3574 (20.8) |
|  | Never | 507 (0.5) | 343 (0.6) | 131 (0.8) |
|  | NA | 23076 (23.0) | 611 (1.1) | 220 (1.3) |
| Fluoride  toothpaste | Not used | 5302 (5.3) | 3889 (6.8) | 1168 (6.8) |
|  | Used | 58798 (58.6) | 43050 (75.3) | 12949 (75.4) |
|  | NA | 36200 (36.1) | 10204 (17.9) | 3050 (17.8) |
| Thumb  sucking habit | No | 67523 (67.3) | 48880 (85.5) | 15277 (89.0) |
|  | Yes | 10490 (10.5) | 8208 (14.4) | 1863 (10.9) |
|  | NA | 22287 (22.2) | 55 (0.1) | 27 (0.2) |
| Teeth  grinding habit | No | 60296 (60.1) | 44087 (77.2) | 13260 (77.2) |
|  | Yes | 17707 (17.7) | 12996 (22.7) | 3882 (22.6) |
|  | NA | 22297 (22.2) | 60 (0.1) | 25 (0.1) |
| Malocclusion | No | 68587 (68.4) | 50049 (87.6) | 15158 (88.3) |
|  | Yes | 9318 (9.3) | 6979 (12.2) | 1958 (11.4) |
|  | NA | 22395 (22.3) | 115 (0.2) | 51 (0.3) |
| Breastfeeding | Never or ceased | 74322 (74.1) | 54743 (95.8) | 16116 (93.9) |
|  | Still breastfed | 1775 (1.8) | 1025 (1.8) | 609 (3.5) |
|  | NA | 24203 (24.1) | 1375 (2.4) | 442 (2.6) |
| Maternal education | Junior high school | 4738 (4.7) | 1658 (2.9) | 896 (5.2) |
|  | High school | 30817 (30.7) | 15515 (27.2) | 6020 (35.1) |
|  | Technical college | 1619 (1.6) | 897 (1.6) | 262 (1.5) |
|  | Vocational school | 22419 (22.4) | 13311 (23.3) | 3784 (22.0) |
|  | Junior college | 17142 (17.1) | 10728 (18.8) | 2869 (16.7) |
|  | University | 19806 (19.7) | 13417 (23.5) | 2950 (17.2) |
|  | Graduate school | 1434 (1.4) | 1004 (1.8) | 198 (1.2) |
|  | NA | 2325 (2.3) | 613 (1.1) | 188 (1.1) |
| Paternal education | Junior high school | 7108 (7.1) | 2984 (5.2) | 1515 (8.8) |
|  | High school | 35808 (35.7) | 18820 (32.9) | 6799 (39.6) |
|  | Technical college | 2108 (2.1) | 1243 (2.2) | 337 (2.0) |
|  | Vocational school | 17807 (17.8) | 10654 (18.6) | 3056 (17.8) |
|  | Junior college | 1995 (2.0) | 1237 (2.2) | 322 (1.9) |
|  | University | 28135 (28.1) | 18221 (31.9) | 4279 (24.9) |
|  | Graduate school | 4392 (4.4) | 3132 (5.5) | 556 (3.2) |
|  | NA | 2947 (2.9) | 852 (1.5) | 303 (1.8) |
| Maternal smoking habit | Never smoked | 56446 (56.3) | 35340 (61.8) | 9276 (54.0) |
|  | Smoked, but quit | 36812 (36.7) | 19529 (34.2) | 6737 (39.2) |
|  | Still smoke | 4466 (4.5) | 1557 (2.7) | 914 (5.3) |
|  | NA | 2576 (2.6) | 717 (1.3) | 240 (1.4) |
| Paternal smoking habit | Never smoked | 26421 (26.3) | 17160 (30.0) | 4073 (23.7) |
|  | Smoked, but quit | 25193 (25.1) | 15856 (27.7) | 4013 (23.4) |
|  | Still smoke | 45093 (45.0) | 22994 (40.2) | 8667 (50.5) |
|  | NA | 3593 (3.6) | 1133 (2.0) | 414 (2.4) |
| Family income | <2 | 5183 (5.2) | 2218 (3.9) | 1091 (6.4) |
| (million yen per year) | 2-4 | 31592 (31.5) | 17248 (30.2) | 5823 (33.9) |
|  | 4-6 | 30234 (30.1) | 18212 (31.9) | 5137 (29.9) |
|  | 6-8 | 14581 (14.5) | 9345 (16.4) | 2249 (13.1) |
|  | 8-10 | 5998 (6.0) | 3814 (6.7) | 966 (5.6) |
|  | 10-12 | 2217 (2.2) | 1448 (2.5) | 345 (2.0) |
|  | 12-15 | 873 (0.9) | 543 (1.0) | 144 (0.8) |
|  | 15-20 | 515 (0.5) | 314 (0.5) | 96 (0.6) |
|  | >20 | 311 (0.3) | 174 (0.3) | 56 (0.3) |
|  | NA | 8796 (8.8) | 3827 (6.7) | 1260 (7.3) |
| Gender | Male | 51394 (51.2) | 28879 (50.5) | 9148 (53.3) |
|  | Female | 48888 (48.7) | 28264 (49.5) | 8019 (46.7) |
|  | NA | 18 (0.0) | 0 (0.0) | 0 (0.0) |
| Gestational weeks (weeks) |  | 39.0 [2.0] | 39.0 [2.0] | 39.0 [2.0] |
| Body mass index (kg•m^-2^) |  | 15.6 [1.4] | 15.6 [1.4] | 15.6 [1.4] |

All variables except for gestational weeks and body mass index are expressed as number (%). Gestational weeks and body mass index are expressed as median [interquartile range].

NA: no answer

Additional Table 3. Baseline characteristics of the participants after excluding cases with neurodevelopmental disorders or neoplastic diseases (n = 72,655).

|  |  | Overall | Caries (-) | Caries (+) |
| --- | --- | --- | --- | --- |
| n |  | 72655 | 55805 | 16850 |
| Number of  brushings  (per day) | None | 132 (0.2) | 91 (0.2) | 41 (0.2) |
|  | 1 | 17456 (24.0) | 13258 (23.8) | 4198 (24.9) |
|  | 2 | 34865 (48.0) | 26584 (47.6) | 8281 (49.1) |
|  | 3 | 19897 (27.4) | 15627 (28.0) | 4270 (25.3) |
|  | >4 | 266 (0.4) | 218 (0.4) | 48 (0.3) |
|  | NA | 39 (0.1) | 27 (0.0) | 12 (0.1) |
| Parental  brushing | Every time | 59158 (81.4) | 46158 (82.7) | 13000 (77.2) |
|  | Occasionally | 12239 (16.8) | 8726 (15.6) | 3513 (20.8) |
|  | Never | 456 (0.6) | 333 (0.6) | 123 (0.7) |
|  | NA | 802 (1.1) | 588 (1.1) | 214 (1.3) |
| Fluoride  toothpaste | Not used | 4944 (6.8) | 3802 (6.8) | 1142 (6.8) |
|  | Used | 54882 (75.5) | 42144 (75.5) | 12738 (75.6) |
|  | NA | 12829 (17.7) | 9859 (17.7) | 2970 (17.6) |
| Thumb  sucking habit | No | 62840 (86.5) | 47829 (85.7) | 15011 (89.1) |
|  | Yes | 9735 (13.4) | 7923 (14.2) | 1812 (10.8) |
|  | NA | 80 (0.1) | 53 (0.1) | 27 (0.2) |
| Teeth  grinding habit | No | 56209 (77.4) | 43176 (77.4) | 13033 (77.3) |
|  | Yes | 16365 (22.5) | 12572 (22.5) | 3793 (22.5) |
|  | NA | 81 (0.1) | 57 (0.1) | 24 (0.1) |
| Malocclusion | No | 63779 (87.8) | 48892 (87.6) | 14887 (88.4) |
|  | Yes | 8714 (12.0) | 6801 (12.2) | 1913 (11.4) |
|  | NA | 162 (0.2) | 112 (0.2) | 50 (0.3) |
| Breastfeeding | Never or ceased | 69291 (95.4) | 53469 (95.8) | 15822 (93.9) |
|  | Still breastfed | 1593 (2.2) | 1002 (1.8) | 591 (3.5) |
|  | NA | 1771 (2.4) | 1334 (2.4) | 437 (2.6) |
| Maternal education | Junior high school | 2482 (3.4) | 1614 (2.9) | 868 (5.2) |
|  | High school | 21025 (28.9) | 15124 (27.1) | 5901 (35.0) |
|  | Technical college | 1135 (1.6) | 876 (1.6) | 259 (1.5) |
|  | Vocational school | 16726 (23.0) | 13005 (23.3) | 3721 (22.1) |
|  | Junior college | 13306 (18.3) | 10487 (18.8) | 2819 (16.7) |
|  | University | 16025 (22.1) | 13124 (23.5) | 2901 (17.2) |
|  | Graduate school | 1178 (1.6) | 983 (1.8) | 195 (1.2) |
|  | NA | 778 (1.1) | 592 (1.1) | 186 (1.1) |
| Paternal education | Junior high school | 4377 (6.0) | 2899 (5.2) | 1478 (8.8) |
|  | High school | 25048 (34.5) | 18368 (32.9) | 6680 (39.6) |
|  | Technical college | 1554 (2.1) | 1220 (2.2) | 334 (2.0) |
|  | Vocational school | 13411 (18.5) | 10424 (18.7) | 2987 (17.7) |
|  | Junior college | 1527 (2.1) | 1210 (2.2) | 317 (1.9) |
|  | University | 22013 (30.3) | 17800 (31.9) | 4213 (25.0) |
|  | Graduate school | 3601 (5.0) | 3055 (5.5) | 546 (3.2) |
|  | NA | 1124 (1.5) | 829 (1.5) | 295 (1.8) |
| Maternal smoking habit | Never smoked | 43672 (60.1) | 34559 (61.9) | 9113 (54.1) |
|  | Smoked, but quit | 25646 (35.3) | 19039 (34.1) | 6607 (39.2) |
|  | Still smoke | 2404 (3.3) | 1512 (2.7) | 892 (5.3) |
|  | NA | 933 (1.3) | 695 (1.2) | 238 (1.4) |
| Paternal smoking habit | Never smoked | 20727 (28.5) | 16735 (30.0) | 3992 (23.7) |
|  | Smoked, but quit | 19429 (26.7) | 15495 (27.8) | 3934 (23.3) |
|  | Still smoke | 30991 (42.7) | 22473 (40.3) | 8518 (50.6) |
|  | NA | 1508 (2.1) | 1102 (2.0) | 406 (2.4) |
| Family income | <2 | 3209 (4.4) | 2151 (3.9) | 1058 (6.3) |
| (million yen per year) | 2-4 | 22490 (31.0) | 16783 (30.1) | 5707 (33.9) |
|  | 4-6 | 22865 (31.5) | 17813 (31.9) | 5052 (30.0) |
|  | 6-8 | 11365 (15.6) | 9157 (16.4) | 2208 (13.1) |
|  | 8-10 | 4678 (6.4) | 3732 (6.7) | 946 (5.6) |
|  | 10-12 | 1754 (2.4) | 1411 (2.5) | 343 (2.0) |
|  | 12-15 | 677 (0.9) | 534 (1.0) | 143 (0.8) |
|  | 15-20 | 404 (0.6) | 309 (0.6) | 95 (0.6) |
|  | >20 | 229 (0.3) | 173 (0.3) | 56 (0.3) |
|  | NA | 4984 (6.9) | 3742 (6.7) | 1242 (7.4) |
| Gender | Male | 36842 (50.7) | 27915 (50.0) | 8927 (53.0) |
|  | Female | 35813 (49.3) | 27890 (50.0) | 7923 (47.0) |
| Gestational weeks (weeks) |  | 39.0 [2.0] | 39.0 [2.0] | 39.0 [2.0] |
| Body mass index (kg•m^-2^) |  | 15.6 [1.4] | 15.6 [1.4] | 15.6 [1.4] |

All variables except for gestational weeks and body mass index are expressed as number (%). Gestational weeks and body mass index are expressed as median [interquartile range].

NA: no answer
